# Knee problems are common in young adults and associated with physical activity and not obesity: the findings of a cross-sectional survey in a university cohort

**DOI:** 10.1186/s12891-019-2487-2

**Published:** 2019-03-18

**Authors:** Chukwuemeka Ibeachu, James Selfe, Chris J. Sutton, Paola Dey

**Affiliations:** 1 School of Medicine, University of Central Lancashire, Preston, PR1 2HE UK; 2 Department of Health Professions, Manchester Metropolitan University, Manchester, UK; 3 Biostatistics Group, School of Health Sciences, University of Manchester, Manchester, UK; 4 Faculty of Health and Social Care, Edge Hill University, Ormskirk, Lancashire UK

**Keywords:** Knee pain, Obesity, Physical activity, Cross-sectional

## Abstract

**Background:**

Obesity and sedentary behaviour, risk factors for knee osteoarthritis in middle-age, are increasing in younger adults. The objectives of this study were to estimate the prevalence of knee problems in young adults, to characterise these problems and explore the relationship with physical activity, physical inactivity and obesity.

**Methods:**

Presence of knee problems was collected through self-report questionnaire from staff and students of one university aged 18–39; direct measurement of weight and height was taken and activity measured using the International Physical Activity Questionnaire. Twelve-month prevalence of knee problems was estimated. Logistic regression was used to investigate the relationship between knee problems and physical activity levels, sitting time and body mass index.

**Results:**

The prevalence of knee problems was high (31.8% [95% CI 26.9 to 37.2%]) among the 314 participants; knee pain was the most common dominant symptom (65%). Only high physical activity levels (OR 2.6 [95% CI 1.4–4.9]) and mental distress (OR 2.3 [95% CI 1.2–4.6]) were independent risk factors for knee problems.

**Conclusions:**

Knee problems were common among young adults, who were staff and students of a university. With increasing obesity prevalence, populations are being encouraged to become more active. More attention may need to be paid towards prevention of knee problems in such programmes, and further research is warranted.

## Background

There has been considerable study of knee pain and its risk factors in middle-aged and older adults, because of the burden of osteoarthritis, particularly in developed countries [1]. In younger adults, academic interest has mainly focused on the epidemiology of specific conditions, such as, patellofemoral pain or cartilage and ligament injury [2–4]. Researchers have also focused mainly on specific populations, such as, the military or athletes [5]. This may be because of the impact of knee pain on performance in elite sports and military training, as well as, an easily accessible population for study. However, established risk factors associated with the development of knee osteoarthritis, such as obesity, are becoming more common in younger populations [6]. Consequently, there is an increasing emphasis on promoting physical activity among the population to reduce obesity-related co-morbidities, such as, heart disease, diabetes and some cancers, but increased activity has been associated with knee injury [7]. A study by Gelber et al. suggested a long-term increased risk of osteoarthritis following a knee injury in young adulthood, which doubled in heavier participants [8]. However, a recent systematic review suggests that the evidence for sport participation and physical activity as risk factors for osteoarthritis is inconclusive [9].

A focus on knee injury potentially underplays the potential role of other knee conditions. There is growing evidence that patellofemoral pain in young adulthood may increase the risk of patellofemoral osteoarthritis in later life [10]. Furthermore, a focus just on knee pain may not encompass the full range of problems experienced by younger people, as locking or giving way may also be symptoms of a knee problem: Felson et al. (2007) found that knee buckling, in the absence of knee pain, occurred in 2.1% of men and women aged 36 to 94 years old [11].

Therefore, it is important to understand the epidemiology of knee problems in more diverse younger populations, as well as, its relationship with obesity, physical activity and physical inactivity. The objectives of this study were to determine the prevalence of knee problems in young adults, to characterise these problems and to explore the relationship between knee problems and physical activity, physical inactivity and obesity.

## Methods

Adults aged between 18 years and 39 years (inclusive) were recruited from staff and students at the University of Central Lancashire, Preston, United Kingdom. Potential participants with doctor diagnosed lower limb osteoarthritis, inflammatory arthritis, or other disorders, which severely affected their walking were excluded. Participants were recruited either as individuals, through advertisement, university media or distribution of leaflets, or as groups (clusters), via classroom sessions. Those willing to participate were provided with an information sheet and checked for eligibility. Once eligibility was confirmed, informed consent was obtained and participants were asked to self-complete a questionnaire that included questions on age and gender, as well as, a number of validated questions/questionnaires. Presence of knee problems was measured using a modification of the screening question in the Knee Pain Screening Tool (KNEST) questionnaire: *“Have you had pain or problems in the last year in or around the knee?”* which has been used in population studies of adults over 50 years of age [12]. In the original study of the measurement properties of the KNEST questionnaire, the knee pain screening question exhibited high internal reliability (R = 0.82 (95% CI 0.66–0.97) and high agreement with a pain mannikin (95%) [12]. The characteristics of the knee problem was assessed using the Survey Instrument for Natural History and Aetiology of Patellofemoral Pain Studies (SNAPPS) [5], which includes questions on dominant symptoms and how the problem developed. The severity of symptoms in those with knee problems was measured by the Knee Injury and Osteoarthritis Outcome Score (KOOS) (http://www.koos.nu), which has a total score and five subscales: pain (nine items), symptoms (seven items), function in daily living (17 items), function in sport & recreation (five items), and quality of life (four items) [13, 14]. Each subscale score is based on the sum of its items transformed into a 0–100 score, with 100 representing ‘no problems’ and zero representing ‘extreme problems’. The Hopkins Symptom Checklist 10 item scale (HSCL-10) was included to measure levels of mental distress in all participants; each of the 10 items in HSCL-10 is scored on a Likert scale of one (not at all) to four (extremely) and the higher the mean across the 10 items the greater the mental distress [15]. The International Physical Activity Questionnaire Long was included to measure levels of physical activity using the questions on activity in the last seven days and physical inactivity, using the questions on sitting time [16]. Height and weight were measured directly, according to an agreed, standard protocol by one researcher (first author) using a Seca 217 mobile stadiometer and Seca 813 scales; however, because it was anticipated that some participants may feel uncomfortable and refuse to be measured, self-reported weight and height was also collected in the questionnaire, which was completed before direct measurement. The study was approved by the University of Central Lancashire Science, Technology, Engineering and Medicine (STEM 025) Ethics Committee.

### Sample size

A sample size of 300 was needed to estimate the prevalence of knee problems to within +/− 4.5% with 95% confidence, assuming a true prevalence of 20% or less and assuming there was no clustering effect on the outcome of interest.

### Analysis

Prevalence estimates and 95% confidence intervals (CI) were estimated. The impact of the clustering induced by the nature of the sampling was examined by estimating the inflation factor for prevalence estimates, that is, the robust standard error adjusted for clustering divided by the crude standard error; an inflation factor above 1 was considered indicative of the presence of a clustering effect [17]. Differences between groups were investigated using chi-squared tests and *P*-values < 0.05 were considered statistically significant.

Logistic regression modelling was used to investigate the effects of BMI, physical inactivity, and physical activity levels on the presence of a self-reported knee problem. Physical activity was categorised into high, medium or low using established protocols [16]. BMI was estimated from the directly-measured weight and height values. However, 66 participants did not consent to have direct measurement of height and/or weight to enable an estimate of BMI (kg/m^2^). These missing values were imputed using a linear regression model using self-reported BMI to predict the participant’s directly-measured BMI; the mean (SD) BMI including imputed data was similar to the mean (SD) of the directly measured BMI (24.3 (4.1) versus 24.1 (4.2)). BMI was maintained as a continuous variable (kg/m^2^). As with BMI, physical inactivity was maintained as a continuous variable (number of hours spent sitting/day) and centred to improve the interpretation of models. Age, gender and mental distress were included as potential confounding factors, given evidence from the literature that these may be important influences on levels of pain [18, 19]. In these analyses, HSCL-10 scores were categorised using a recognised cut-off of 1.85 [15]: (i) those with a score less than 1.85 were categorised as having no mental distress and (ii) those with a score greater than or equal to 1.85 categorised as having mental distress. Exploratory analysis was performed to assess the likely nature of the relationship of the continuous variables with the logit of self-reported knee problem; if the nature of the relationship appeared quadratic, both linear and quadratic terms were included in the regression model. The analysis then firstly examined the unadjusted effects of the three putative risk factors individually, and, secondly examined the effects of each putative risk factor, while adjusting for the effects of the other two and the potential confounding factors.

The performance of the models including one, two or all three of the putative risk factors and all three potential confounding factors was compared using areas under the curve of ‘percentage correct predictions’ and the Hosmer-Lemeshow goodness-of-fit test [20]. The optimal model was judged based on maximising the AUC whilst assuring no significant lack of fit (at the 10% level). Additionally, a potential interaction between physical activity and physical inactivity was investigated by adding this term to the previously best-fitting model and testing its significance using a 10% level; this was considered as those who had high levels of both activity and inactivity were thought to be potentially particularly prone to injury. Odds ratio (OR) for univariate models and the optimal multivariate model are reported with 95% CI.

## Results

A total of 314 participants were recruited between January and October 2013. The participant characteristics are shown in Table 1. There were more men than women recruited, and there were relatively few participants over 30 years of age (33, 10.5%), reflecting the low number of staff taking part. Only 47 (15.2%) of the 314 participants had an HSCL-10 score above the cut-off for mental distress, while 31 (9.9%) were obese and 72 (23.2%) were overweight. Mean (SD) total hours spent sitting per day was 5.6 h (SD 2.6) and over half of the participants (165, 52.9%) reported low physical activity with similar proportions reporting moderate (75, 24.0%) and high (72, 23.1%) physical activity levels. The majority (86%) of the participants were recruited from 14 classes; the remaining participants were recruited as individuals. Assuming 15 clusters, there was no evidence of clustering for estimates of period prevalence of knee problems and therefore, statistical methods and confidence intervals relating to this estimate were not adjusted for clustering.

**Table 1:** Characteristics of the 314 participants

	Without knee problem	With knee problem	Overall
	*N* = 214	*N* = 100	*N* = 314
Male gender n (%)	115 (53.7%)	61 (61%)	176 (56.1%)
Mean (SD) age in years	21.9 (5.0)	22.6 (5.4)	22.0 (5.2)
HSCL-10			
n (%) meeting threshold for mental distress^a^	26 (12.4%)	21 (21.0%)	47 (15.2%)
Mean (SD) BMI (kg/m^2^)^b^	24.1 (4.1)	24.8 (4.1)	24.3 (4.1)
Physical activity levels n (%)			
Low	121 (56.5%)	44 (44.0%)	165 (52.5%)
Moderate	50 (23.4%)	25 (25.0%)	75 (23.9%)
High	42 (19.6%)	30 (30.0%)	72 (22.9%)
Missing	1 (0.5%)	1 (1%)	2 (0.6%)
Mean (SD) average daily sitting time (in hours)	5.5 (2.5)	5.9 (2.9)	5.6 (2.6).

^a^5 missing ^b^248 from directly measured height and weight; 63 imputed from self-reported height and weight; 3 missing

A total of 100 participants reported a knee problem in the previous 12 months giving a 12-month period prevalence of 31.8% (95% CI 26.9 to 37.2%), of whom 44 (44%) had bilateral problems and 52 (52%) had previously sought medical advice on the knee problem. Of the 100 participants with knee problems, 31 (31%) recalled that their knee problem developed following a sudden injury. Of the 100 participants with knee problems, 93 provided information about symptoms. Of these 93 participants, pain was the predominant symptom in 65 (69.9%); other symptoms included locking (7/93, 7.5%) and giving way (21/93, 22.5%). Although, daily living activities were not particularly affected in most of the participants with a knee problem (median KOOS score 96), quality of life did appear to be affected (median KOOS score 75) with 30% having a KOOS score for that subscale of less than 60 (Table 2).

**Table 2:** Types and severity of the symptoms of in the 100 participants with knee problems (measured by the KOOS)

Predominant symptom^a^	N (%)
Pain	65 (69.9%)
Locking	7 (7.5%)
Giving way	21 (22.5%)
KOOS subscale	Median (IQR)
Pain	89 (78 to 97)
Severity of symptom	82 (75 to 89)
Function, daily living	96 (89.7 to 100)
Function, sports and recreational activities	90 (67.5 to 100)
Quality of life	75 (56 to 88)

^a^7 missing; *KOOS* Knee injury and osteoarthritis outcome score, *IQR* Inter-quartile range

The characteristics of those participants that reported knee problems and those that did not are shown in Table 1 and compared in Table 3 (univariate analysis). As there was no evidence of an interaction (*p* = 0.83), only the ‘main effects’ of these variables were included in the final regression model. The final regression model suggested that there was an increase in the prevalence of knee problems with each 10 kg/m^2^ increase in BMI, hours of sitting and higher physical activity levels, (Table 3), although only the latter was statistically significant (*p* = 0.010). There was a borderline non-significant difference in mental distress between those that had a knee problem and those that did not; the percentage meeting the threshold for mental distress on the HSCL-10 was 21% (21/100) of those with knee problems and 12.4% of those without knee pain (26/209) (p = 0. 0.052; OR 1.9 95% CI 1.0 to 3.5) (Table 1). In the multivariate analysis, the only significant independent factors related to reported knee problems were physical activity (p = 0.010; high level: OR = 2.6, 95% CI 1.4 to 4.9; moderate level OR = 1.9, 95%CI 1.0 to 3.5) and mental distress (*p* = 0.017; OR = 2.3, 95% CI 1.2 to 4.6). The effects of BMI (*p* = 0.063) and hours of physical inactivity (*p* = 0.069) were borderline non-significant. The Area under the Curve for the best regression model was 0.7 and the Hosmer-Lemeshow goodness-of-fit test was not significant (*p* = 0.16), indicating a satisfactory fit of the model [20].

**Table 3:** Univariate and multivariate logistic regression analysis of the effect of physical activity, BMI and physical inactivity on the prevalence of knee problems adjusting for all other variables

Characteristic	Univariate	Multivariate	Multivariate
	OR (95% CI)	OR (95% CI)	*p*-value
Age / 5 years	1.1 (0.9–1.4)	1.2 (0.9–1.5)	0.25
Gender			
Male	1	1	
Female	0.7 (0.5–1.2)	0.9 (0.5–1.6)	0.74
Mental distress			
< 1.85	1	1	
≥1.85	1.9 (1.0–3.5)	2.3 (1.2–4.6)	0.017
BMI /10 kg/m^2^	1.5 (0.9–2.7)	1.8 (1.0–3.4)	0.063
Physical activity levels			0.010
Low	1	1	
Moderate	1.4 (0.8–2.5)	1.9 (1.0–3.5)	
High	2.0 (1.1–3.5)	2.6 (1.4–4.9)	
Average daily sitting time (in hours)			0.069
Linear term	1.03 (0.93–1.14)	1.04 (0.93–1.16)	
Quadratic term	1.02 (0.99–1.04)	1.02 (1.00–1.05)	

Odds ratio (OR) for knee problems adjusted for the other factors in the table. Body mass index (BMI)

## Discussion

The findings suggest a high prevalence of knee problems in this age group with nearly a third having a knee problem in the last year. The prevalence rate is higher than that observed in a large-scale epidemiological study for similar age groups [21]. However, the focus of that study was on detection of disabling knee conditions and the questions were more stringent in their criteria.

In our study, the observed prevalence of knee problems was higher in males compared with females, but this difference was not statistically significant. Different knee conditions appear to have a predominance in different genders. For example, females have a higher rate of patellofemoral pain [22]; others have found soft tissue injuries to be more common in males with the lowest rates in females to be in those aged 20 to 39 years [23]. The gender predominance may largely balance out when considering all knee conditions.

Overall, our study suggests that focusing on specific conditions or just on knee pain may underestimate the problem of musculoskeletal disorders within younger populations; and that knee problems may be a hidden health problem as only half had sought medical advice. This is not an insubstantial issue as it has some important public health implications. There is increasing recognition that osteoarthritis can take decades to develop and that earlier identification of risk factors for osteoarthritis and/or symptoms of early diagnostic stages might offer benefits in terms of prevention and/or reduction in disability [8, 23, 24]. Buckling is an important symptom in older populations in whom it has been associated with activity limitation; in our study, 22.5% of those with knee problems had giving way as a predominant symptom [25]. The numbers were too small to investigate whether function, quality of life or risk factors differed in different types of knee problem. We found that only physical activity was independently associated with knee problems. Others have noted a relationship in adolescents [26]. The best evidence for a risk factor for osteoarthritis that affects younger people is for obesity [9], and this is of particular public health concern given the increased prevalence in younger adults [6]. In this study, although the estimated odds of a knee problem almost doubled for each 10 kg/m^2^ increase in BMI, the relationship between BMI and presence of a knee problem was of borderline non-significance. While it might be that the study was underpowered to detect a difference, the average age of the study population was only 22 years old and it could be that this is too early for the structural changes in the knee associated with obesity to manifest as symptoms.

Increased physical activity was associated with increasing risk of knee problems in this population and this relationship appeared linear, with increasing risk with each increase in level of activity. Although, the evidence for physical activity as a risk factor for osteoarthritis may be more limited [9, 27], these findings raise opposing public health concerns. Low levels of physical activity are associated with obesity and co-morbidities and, consequently, increasing physical activity is recommended by health organisations to prevent obesity, heart disease and diabetes. More recently there is increasing interest in high intensity interval training to improve health outcomes in less active populations [28]. But there has been little work undertaken on musculoskeletal injury in naïve exercisers.

There was also a significant relationship between mental distress and presence of a knee problem. This needs to be investigated in incidence studies as it is unclear if mental distress precedes or follows the development of the problem. Nevertheless, those with knee problems in this study had much worse mean quality of life scores on the KOOS (median 75) compared with a similar healthy population age group (median 100) suggesting considerable psychosocial burden [29]. Psychological morbidity has been observed among those not returning to pre-level sports participation following sports injury and in patellofemoral pain, where there is some relationship with pain levels and function [18, 30]. Further work on the economic burden of knee problems in young adults is warranted.

### Study limitations

An important limitation of this study is its cross-sectional design. The problem of a lack of information about the direction of the effect is highlighted above in relation to mental distress. In addition, studies have shown that obese adults are less likely to be active [31]. It might be that those who have knee problems and are obese are more likely to reduce the activities which bring on symptoms and, therefore, would not have had the problem at the time of the survey. However, the findings suggest that further large-scale incidence studies are warranted.

The University had 2666 and 26,585 staff and students respectively at the time of the study, but data was not available as to their characteristics to identify the proportion who would meet the eligibility criteria and a sampling framework was not available to facilitate random selection of participants. Therefore, a convenience sample of staff and students of the university was used in the selection of participants recruiting until the sample size was obtained. The population group made it feasible to achieve the target sample size. However, this sampling method could have introduced selection bias. For example, those with a history of knee problems may have been more likely to consent to participate, thus potentially overestimating the prevalence of knee problems, but we emphasised that we needed participants who did not have knee problems as well as those who did in our recruitment strategies. Another concern is that participants may be more active than the general population because a third were on courses linked to activity and, hence, more prone to knee problems, given our findings that physical activity was associated with knee problems. Conversely, though, a university cohort is less likely to undertake manual work. Reassuringly, we found a similar prevalence rate of 33% in a general population sample of adults of the same age attending a science festival using the same question [5]. The question we modified had been validated to screen for knee pain in adults over 50 years of age [12]. While it has not been formally evaluated in younger people, the prevalence rate is not dissimilar to that observed by Rathleff using a pain mannequin in a population-based study of adolescents [32].

## Conclusion

In conclusion, knee problems were common in young adults in a university and this appears to be associated with high physical activity levels. Populations are being encouraged to become more active. More attention may need to be paid towards prevention of knee problems in such programmes, and further research is warranted to investigate, in large-scale, population-based studies, if these findings are generalisable to general population and across different types of knee symptoms.

## Abbreviations

AUC: Area under curve
BMI: Body mass index
CI: Confidence interval
HSCL- 10: Hopkins symptom checklist 10 item scale
KNEST: Knee Pain Screening Tool
KOOS: Knee injury and osteoarthritis outcome score
OR: Odd ratio
SD: Standard deviation
SNAPPS: Survey instrument for natural history and Aetiology of Patellofemoral pain studies
